# Short-term effectiveness of three agents for dentin hypersensitivity treatment: A randomized clinical trial

**DOI:** 10.6026/973206300222206

**Published:** 2026-04-30

**Authors:** Ankita Garg, Mohit Garg, Mohan Rawat

**Affiliations:** 1 Department of Dentistry, Shyam Shah Medical College, Rewa, Madhya Pradesh, India; 2 Department of Periodontology, K.D. Dental College and Hospital, Mathura, Uttar Pradesh, India

**Keywords:** Dentin hypersensitivity (DH), acidulated phosphate fluoride (APF) gel, iontophoresis, GLUMA, Clinpro XT, visual analog scale (VAS) score

## Abstract

Dentin hypersensitivity is common and many desensitizing agents exist, but comparative data across products and delivery methods remain limited. Therefore, it is of interest to compare three interventions-GLUMA Desensitizer, Clinpro XT Fluoride Varnish and 1.23% APF gel with iontophoresis applied in 75 patients using two stimuli (cold air blast and explorer scratching) and assessed by VAS at six time points up to 120 days. All three groups showed significant reductions in hypersensitivity over time on both stimuli, with the greatest VAS decline seen in the APF-iontophoresis group, particularly under air blast stimulation (mean change 63.28 ±12.80; p < 0.001). No significant intergroup differences were observed with explorer scratching, suggesting stimulus-dependent discrimination of efficacy. Thus, we show that 1.23% APF gel with iontophoresis provides the most sustained and clinically meaningful relief, supporting its preferential use for long-term management of air-induced dentin hypersensitivity.

## Background:

Dentinal hypersensitivity (DH) is the most common dental condition and in practice, remains underappreciated. Since it is prevalent, it is one of the important conditions to be addressed [1]. It is a condition of temporary sharp pain from open dentinal surfaces to outside stimuli that are usually thermal, tactile, osmotic, evaporative, or chemical [2]. It is not only a painful but also a disabling condition because of its negative effect on oral health-related quality of life [3, 4]. Dentinal hypersensitivity is not an emergent, life-threatening disease. Still, the repeated discomfort it causes can significantly affect food choices, oral health habits and the patient's overall well-being [4]. The DH pain has been classically described as a response that another oral disease cannot explain. The most widely used definition came from the Canadian Consensus Document, which defined DH as "pain resulting from exposed dentine secondary to chemical, thermal, tactile, osmotic, or evaporative stimulation which cannot be attributed to any other dental flaw or pathology [5]. It requires an exclusion diagnosis, since the clinical situation could also be caused by other pathologies such as cracked tooth syndrome, broken restorations, caries, or pulpal inflammation [6, 7- 8]. Adequate diagnosis, therefore, forms the cornerstone for appropriate management [8]. Dentin exposure is a mandatory prerequisite for DH to take place and a host of etiologic factors can initiate this [9] Enamel or cementum structural loss, due to abrasion, attrition, erosion, or abfraction, weakens the tooth's protective layer, leading to dentin exposure. Furthermore, periodontal infection-induced gingival recession exposes root surfaces; making one more susceptible to DH [10]. Cements with a thin film over root dentin are susceptible to removal or absence, creating an open pathway for transmission of the stimulus to the pulp [11]. Permeability and patency of dentinal tubules are most important for the cause and severity of DH. Histologically, it was confirmed that hypersensitive dentin consisted of much more patent dentinal tubules with a larger diameter than non-sensitive dentin. A few of the factors regulating patency of tubules are the presence or absence of a smear layer, the extent of peritubular sclerosis of dentin and the amount of secondary or reparative dentin pulpal to it [12, 13]. Physiological aging leads to dentinal changes such as sclerosis and secondary dentin deposition, reducing the permeability and being involved in the decreased incidence of hypersensitivity in aged populations [14]. Epidemiologically, the prevalence of dentinal hypersensitivity varies widely, ranging from 3% to 57% in the literature, with factors including research design, study population and diagnostic criteria [14]. It is most common in adults aged 20-40 years, but may be found in any age group. It has been noted to be more common in females. It may be driven by behavioral and physiological factors, as well as the greater female drive to care for their teeth through visits to dental clinics [15]. Root sensitivity, a form of dentinal hypersensitivity due to gingival recession, has been noted to increase following periodontal treatment, with incidences reported as 9-23% pretherapy and 54-55% post therapy [16]. The same is usually transient and subsides within 3 weeks of treatment. Dentinal hypersensitivity has been the subject of considerable scientific attention aimed at understanding its mechanisms [16, 17]. Among several proposed theories, such as the neural modulation theory, gate control theory and vibration theory, the most scientifically sound and widely adopted is Brännström's hydrodynamic theory. On this premise, pressure from external stimulation forces fluid relatively quickly along the dentinal tubules [18]. Fluid movement changes pressure conditions, activates mechanoreceptors associated with Aδ fibers and produces typical acute pain. The effectiveness of this mechanism depends on the openness of dentinal tubules at both the outer dentin surface and directed away from it towards the pulp [19]. Owing to its multifactorial etiology, DH is best treated with a sympathetic, individualized approach. Before therapy is started, caution should be exercised to ensure that the diagnosis has been established and that differential diagnoses such as carious lesions, pulpitis, tooth chipping, or faulty restorations have been Untreated pathology can cause not only persistent symptoms but also reduce the efficacy of therapeutic intervention intended excluded [15, 18 and 20]. Treatment modalities for dentinal hypersensitivity have exhibited marked variability over the years. They may be generally divided based on mode of action into two groups: (1) neural desensitization and (2) occlusion of dentinal tubules [21]. The first includes actions of agents such as potassium nitrate, which interfere with the transmission of the nerve impulse and the second includes open dentinal tubule physical or chemical occlusion to stop fluid flow [21]. The occlusion mechanism diverges into three mechanisms that facilitate surface-barrier formation (i.e., particulate deposition in toothpaste) and those that result from in-place mineralization (i.e., precipitation of calcium- phosphate). Modalities may also be separated by delivery modes, like use at home, self-applied products, versus in-office professional use [22]. Home treatment generally consists of desensitizing toothpaste and mouthwashes with active agents such as potassium nitrate, sodium fluoride, stannous fluoride, or strontium chloride. They are easy to apply for patient compliance, but take 2 to 4 weeks to show symptom improvement [23]. Office therapies will have more immediate and prolonged effects and are typically reserved for refractory or severe cases [23]. Among office therapies, adhesive restorative materials, dentinal bonding agents and desensitizing varnishes have been encouraging. They act by sealing dentinal tubules, thereby interfering with the hydrodynamic mechanism of pain stimulation. For example, Gluma Desensitizer, an adhesive resin matrix containing hydroxyethyl methacrylate (HEMA), glutaraldehyde, benzalkonium chloride and fluoride, has become widely used because it can provide short-term and long-term relief [24]. Glutaraldehyde is used to induce coagulation of tubular proteins and HEMA is used for infiltration and sealing with the resin. Another great in-office choice is the use of fluoride varnishes, such as Clinpro XT, a resin-modified glass ionomer that not only bonds to the tooth surface but also releases calcium, fluoride and phosphate ions [25]. These ingredients help form insoluble calcium fluoride globules that occlude dentinal tubules, thereby creating a transient physical barrier and a prolonged chemical barrier. Clinpro XT is also augmented by the fact that it can be recharged by fluoride during routine tooth brushing, thus prolonging its curative action [24, 25]. Semi-invasive methods such as iontophoresis have also been reported to be cost-effective treatment modalities in patients characterized by extreme hypersensitivity [25]. The method employs low-voltage electrical current to force ionized desensitizing chemicals, such as 1.23% acidulated phosphate fluoride (APF) gel, into dentinal tubules. Iontophoresis, aside from improving and enhancing the penetration of fluoride ions, also facilitates micro-precipitation of calcium fluoride, reparative dentinogenesis and even re-shaping paresthesia that is conductive. All the synergistic effects provide immediate and typically permanent relief from pain. Increased availability of desensitizing agents has prompted comparative efficacy assessment to define relative efficacy among the variety of materials [26]. Despite the prevalence of several preparations, a broad lack of head-to-head clinical data demonstrating clear superiority among them remains. Thus, methodologically rigorous comparative research needs to provide the data that clinical decision-making and evidence-based treatment planning demand [27]. The choice of an appropriate desensitizing agent, in addition to the size and severity of the lesion, should also take into account patient preference, oral hygiene habits and available compliance. Therefore, it is of interest to evaluate the efficacy of three common treatments for dentinal hypersensitivity: GLUMA Desensitizer, Clinpro XT Fluoride Varnish and 1.23% APF Gel Iontophoresis.

## Materials and Methodology:

## Study design:

It was a prospective, randomized, single-center, parallel-arm clinical trial comparing and assessing the effectiveness of three desensitizing agents-GLUMA Desensitizer, Clinpro XT Fluoride Varnish and 1.23% APF Gel with iontophoresis in managing dentinal hypersensitivity. The study population consisted of 75 otherwise healthy individuals (40 males and 35 females), aged 18 to 65 years. Patients were randomly assigned to three groups (25 per group). The study was planned in accordance with the research ethics guidelines of the institution's research committee and written informed consent was obtained from all participants following adequate explanations of the research protocol, potential discomforts, harms and benefits. Participants were recruited within three months and follow-up data were obtained 120 days later. This strict design allowed real-time monitoring and careful assessment of short- and long-term consequences, as well as of the tested desensitizing products.

## Study setting:

The study was conducted in a clinical dental office with standardized equipment and facilities to enable standardized patient assessment and material application. The subjects were selected from patients attending the outpatient department for dental consultation. Baseline assessment, intervention and post-intervention assessment were conducted from January to June, including recruitment, intervention administration and follow-up measurement. The subjects were measured on six occasions: baseline (day 0), post-intervention, day 14, day 28, day 90 and day 120.

## Study population:

The population studied comprised 75 otherwise healthy individuals, 40 men and 35 women, aged 18-65 years, with a mean age of 39 years. Entry was restricted solely to the presence of clinically verified dentinal hypersensitivity due to typical symptomatology of instantaneous stinging pain originating from the buccal cervical area of teeth on stimulation by external stimuli, like manual explorer scratch test and cold air. Patients with clinical evidence of gingival recession, cervical abrasion, erosion, or any combination of these and who had at least 2 sensitive teeth as determined by clinical examination, were included. Excluded were participants with any dental condition that may simulate symptoms of dentinal hypersensitivity, such as caries, cracked tooth syndrome, defective restorations or pulpitis. Participants undergoing orthodontic treatment, removable prosthetic appliances, a history of allergy to any test agent, regular consumption of analgesics, or those with mental illnesses that would invalidate pain assessment, were excluded from the study.

## Screening and diagnostic tests:

All patients received a complete intraoral examination, including both clinical and radiographic assessments, to rule out confounding dental disease. Dentinal sensitivity was quantified and verified by two diagnostic stimuli: (1) a 3-second discharge of cold air from a dental syringe at 40-60 psi and around 19 ± 3°C onto the cervical surface of the tooth from 3 mm and (2) a tactile test with a dental explorer set perpendicular to the open dentinal surface with a force of 25g. All the teeth were separated using cotton rolls and a sheet of cellophane to prevent any error in the application of stimulation.

Pain sensitivity was assessed through the use of a 4-point subjective scale:

[1] 0 = No pain

[2] 1 = Small pain

[3] 2 = Pain on stimulation alone

[4] 3 = Pain on and following stimulation

The repeated-stimulus effect was prevented by ensuring a minimum of 5 minutes between applications. Moreover, a Visual Analogue Scale (VAS) was used to assess each subject's overall perception of dentinal sensitivity. It was anchored with labels and contained a 10 cm horizontal line with "no pain" at 0 cm and "severe pain" at 10 cm. The participants marked the scale to indicate their level of discomfort and the reading was recorded in millimeters (0-100 range), enabling quantification of the subjective pain experience.

## Randomization and group allocation:

75 participants were randomly assigned to groups of equal size (n = 25 each) using a computer-generated list of randomization:

[1] Group 1: GLUMA Desensitizer

[2] Group 2: Clinpro XT Fluoride Varnish

[3] Group 3: 1.23% APF Gel with Iontophoresis

The allocation was hidden in sequentially numbered, sealed, opaque envelopes and interventions were administered by a seasoned clinician blinded to the grouping at baseline measurement.

## Application of interventions:

For GLUMA Desensitizer Group 1, cotton rolls were used to isolate the affected teeth and the tooth surfaces were gently dried. A small amount of GLUMA was brushed on the exposed cervical dentine with a microbrush. Air from a three-way syringe was used to facilitate the material's absorption. If symptoms were still present upon re-evaluation, treatment was repeated. In Group 2 (Clinpro XT Varnish), the teeth were etched individually with 37% phosphoric acid for 10 seconds and air-dried lightly to avoid moisture contamination. The two- paste varnish was mixed and applied thinly to the surface, which was then light-cured for 20 seconds. Follow-up of the treated area for symptom remission was performed after application. The application process in Group 3 (1.23% APF Gel Iontophoresis) was more involved. The teeth were isolated and dried. APF gel was loaded into a sponge applicator, which was immediately positioned over the sensitive teeth. A foam tray on the cathode (black electrode) of the iontophoresis device (Jonofluor Praxis Master ®) was positioned over the sponge. A current of 2.5 mA was applied for 1 minute and the gel was reapplied to ensure proper penetration and tubule occlusion.

## Variables and data collection:

The reduction in dentinal hypersensitivity was the main outcome measure; assessed using the stimulus-response scale (0-3) and a VAS score (0-100 mm). Baseline values were compared with the values measured immediately after treatment and on days 14, 28, 90 and 120. Gingival recession (in millimeters), abrasion grades and enamel loss were secondary variables documented. To control variability and reduce inter-examiner variability, the same examiner performed all sensitivity testing using standardized stimuli and measurement techniques. Gingival recession was measured using a calibrated dental probe (William's probe) and cervical abrasion was scored according to an agreed 4-point scale.

## Bias control measures:

The study used several strategies to reduce bias. Selection bias was minimized using randomization and allocation concealment. Detection risk was minimized using evaluator blinding of treatment groups. Measurement bias was minimized using standardized protocols and instruments for all tests and materials used. Repeat measurements at multiple time points maximized internal validity and enabled temporal assessment of the treatment's effectiveness.

## Sample size justification:

The sample size of 25 participants per intervention group was chosen following previous research reporting medium-to-large effect sizes for the decrease in dentinal hypersensitivity following topical application of desensitizing agents. Assuming a power of 80% and alpha = 0.05, at least 20 participants in each intervention group were required to detect statistically significant differences between the interventions. As attrition or dropouts of some kind are bound to occur, 5 additional participants were included in each group.

## Treatment of quantitative variables:

All outcome measures were treated as continuous variables for statistical analysis. The VAS scores were presented as mean ± standard deviation. The data were also compared across time points to identify trends in symptom relief. Tactile and air-stimulus scores were treated as ordinal data and presented as medians with interquartile ranges where appropriate.

## Statistical analysis:

Descriptive statistics were calculated for all baseline measures. Intra-group comparisons between time points were analyzed using paired t-tests or Wilcoxon signed-rank tests, depending on the data distribution. Inter-group comparisons were analyzed using one-way ANOVA or Kruskal-Wallis tests and post-hoc Bonferroni or Dunn's tests were performed where required. Repeated-measures ANOVA were used to assess changes in VAS scores over time points within and between groups. A p-value of <0.05 was used for statistical significance.

## Results:

The present study is a parallel-group, single-center, randomized trial comparing three desensitizing agents. Of this number, 75 patients were originally enrolled and all were confirmed to meet the inclusion criteria and agree to the intervention. It attempted to compare the efficacy of two desensitization methods-the Air Blast Method and the Explorer Scratching Method across several agents at six time points: baseline, 1 day, 14 days, 28 days, 90 days and 120 days. Visual Analog Scale (VAS) scores were employed as the primary outcome measure to assess decreased sensitivity. Within-group analysis in all six groups, baseline mean sensitivity scores significantly reduced at subsequent time points, demonstrating the therapeutic effectiveness of each material regardless of the method used. The cold air blast method consistently yielded a greater reduction in VAS scores than the explorer scratch method across all three material groups. For Group 1, the air blast test revealed a decline in VAS scores from baseline 53.60 ±8.32 to 4.60 ±4.14 on day 120. The same group's corresponding explorer scratching test baseline of 54.88 ±7.50 dropped to 5.04 ±5.03 on day 120. ANOVA values were in support of statistical significance (F = 531.52 for air blast, F = 592.257 for explorer scratching, both p < 0.001) (Table 1 - see PDF). In Group 2, the air blast method decreased sensitivity from 59.64 ±9.13 to 3.04 ±3.64 and from 52.72 ±8.35 to 2.76 ±3.68, on day 120 using the explorer scratching technique. These decreases were also statistically significant (F = 560.151 and F = 506.716, respectively, p < 0.001) (Table 1 - see PDF). Group 3 also exhibited the greatest initial sensitivity (66.48 ±12.63 with air blast), which reduced the most, with scores falling to 3.20 ±3.88 at 120 days. Explorer scratching technique in Group 3 also followed the same pattern, falling from 54.56 ±8.55 to 3.00 ±3.96. The statistical comparisons between the two were highly significant (F = 543.101 and F = 645.338, p < 0.001), further supporting the therapeutic action of the material in Group 3 (Table 1 - see PDF). When comparing the reduction in sensitivity scores from baseline to 120 days between the three groups using the air blast method, a significant difference was noted (F = 10.491, p < 0.001). Group 3 (1.23% APF gel + iontophoresis) showed the greatest improvement (mean change: 63.28 ±12.80), significantly outperforming Group 1 and Group 2 in post hoc comparisons (p < 0.001 for Group 3 vs Group 1; p = 0.107 for Group 3 versus Group 2). However, in the explorer scratching method, inter-group differences were not statistically significant (F = 0.277, p = 0.759), suggesting similar performance across all three materials (Table 2-4 - see PDF). Thus, the study confirmed that all three desensitizing agents significantly reduced dentinal hypersensitivity over the 120-day observation period. The cold air blast method yielded consistently superior results across groups compared to the explorer scratching method. Among the three materials, the 1.23% APF gel combined with iontophoresis (Group 3) proved to be the most effective, especially when delivered via the air blast method, showing the largest and statistically significant improvement in sensitivity scores. Although Group 3 also demonstrated the best results under the explorer scratching method, the intergroup differences did not reach statistical significance.

## Discussion:

Dentin hypersensitivity (DH) is a widespread clinical issue characterized by transient, sharp pain from exposed dentin in response to exogenous stimulation, e.g., thermal, tactile, osmotic, or chemical. DH significantly influences oral health-related quality of life. Both professional and non-prescription approaches have been recommended to manage DH. Desensitizing toothpastes, in particular, are a simple, non-invasive and well-received management strategy. This review consolidates the findings of the current study and compares them with recent evidence. All three products: potassium nitrate-based, strontium chloride-based and 1.23% APF gel with iontophoresis exhibited statistically significant reductions in VAS scores at multiple time points between baseline and 120 days. Of these, the 1.23% APF gel with iontophoresis (Group 3) experienced the most significant and sustained reduction in dentinal hypersensitivity, especially using the cold air blast technique. All of these results are consistent with the general rule that iontophoresis enables ionic flow into dentinal tubules, improving occlusion and providing more sustained relief. Comparing the in-group differences, the three groups showed a significant reduction in sensitivity on day 1 and, thereafter, a gradual decline until day 90. Minor spikes in VAS scores were observed at day 120, suggesting the need for reinforcement therapy. Between-group differences from ANOVA and post hoc Bonferroni tests revealed that Group 3 performed significantly better than Groups 1 and 2 under the air-blast modality. On the other hand, a statistically significant difference between groups was not observed with the explorer scratching modality, showing the influence of application modality on clinical outcome. These findings agree with those of Parkinson *et al.* who tested the effect of TP-based toothpaste over four weeks. They used a placebo (Pleasia fluoride-free), a strontium chloride (SC) toothpaste and a TP-based toothpaste (Vussen S) group for their clinical trial [28]. Results showed that Group TP performed significantly better than the negative control under both air-blast and cold stimulation (p = 0.0002 and p = 0.021, respectively) and had a greater reduction in VAS score than Group SC (p = 0.012). This is in line with Group 3's superior performance in the current study. Also, Schiff's sensitivity scores in their study showed significant DH reduction with chronic use across all groups, as did the current study's detection of time-dependent improvement. Jang *et al.* identified no statistically significant group difference in Schiff scores at 4 weeks (p = 1.000), possibly due to the study's shorter 120-day follow-up compared with the current study [27]. Also, the meta- analysis of Parkinson *et al.* reaffirms that tricalcium phosphate (and calcium-based bioactive ceramics) exhibit biomimetic properties [28]. The materials incorporate into the tooth structure, occlude dentinal tubules and facilitate in-situ remineralization. Calcium ion and phosphate ion biocompatibility, as well as occlusal potential, suggest that these products may mimic natural enamel repair. This mechanism is supported by the current study, in which APF gel combined with iontophoresis provided rapid and sustained tubular occlusion. Minkoff *et al.* had previously observed that SC-based toothpastes for tooth desensitization have a therapeutic effect within two weeks and this effect increases with duration, especially with thermal and tactile stimuli [29]. Our study supports this, since Group 2 (strontium chloride) exhibited a dramatic reduction in sensitivity by day 90. However, Group 3 still registered superior long-term effectiveness. The relative literature shows that while SC performs satisfactorily, particularly in the short term, TP-based or fluorocalcium phosphate-based products have a greater and longer-lasting impact on DH. In a double-blind trial by Li *et al.*, a 0.454% stannous fluoride dentifrice provided intense short-term DH relief when used with an advanced focused-brushing mode [30]. While both the control and test groups showed improvement, statistically significant differences in favour of the test dentifrice were seen on Day 14. This would support the idea that, even within the short-term frame, some fluoride products may be better, provided their delivery mode is optimized. Interestingly, iontophoresis-augmented APF gel application in our study might have been a form of targeted delivery that augmented the desensitizing effect. Another important feature of our current study is the lack of a significant rebound in VAS scores at 120 days across all groups Jang *et al.* also referred to placebo and Hawthorne effects in DH trials [28]. A placebo effect of nearly 48% in their negative control group resulted from mechanical occlusion of the tubules by abrasive particles, improved oral hygiene and heightened patient compliance. While such effects could have partly contributed to the initial improvements across all groups in the present study, the long-term reductions, particularly in Group 3, can reasonably be ascribed to the chemical and physical properties of the APF gel used for iontophoresis. Of note, the results of the current study are an addition to a clinical trial that demonstrated better performance of the stannous fluoride-based dentifrice compared to strontium chloride-based dentifrices [29]. Even as fluoride-containing toothpaste, especially with advanced delivery systems, has yielded promising results, long-term clinical trials such as the current one are required to establish its long-term efficacy. Furthermore, bioactive materials such as nano-hydroxyapatite (n-HA), tricalcium phosphate and calcium sodium phosphosilicate have been reported to exhibit anti-inflammatory properties and desensitizing activity. Such effects may be beneficial for patients with both DH and incipient gingivitis. The clinical trial is strengthened by its strict design, random allocation, extended follow-up (120 days) and the application of both tactile and thermal stimuli to mimic real clinical practice. Both explorer scratching and air-blast as application modalities enabled robust evidence on the performance of each desensitizing agent in divergent application modalities. Furthermore, repeated-measures ANOVA and Bonferroni post hoc tests ensured the reliability of the statistical conclusions. However, the study has some limitations. While efforts were made to keep things consistent, the natural variation in patients' compliance with home oral hygiene could have affected the results. Second, while efficacy in Group 3 was enhanced by iontophoresis, the method and device aren't necessarily standard equipment in every dental office, limiting generalizability. Lastly, although the exclusion of fluoride-containing pastes was implemented, environmental exposure to fluoride (e.g., fluoridated drinking water) could not be fully eliminated, which could have affected remineralization results.

## Conclusion:

We show that the three desensitizing products tested are beneficial for relieving DH and that the 1.23% APF gels with iontophoresis delivers the most intense and sustained relief. When taken together with existing literature, these findings validate the inclusion of iontophoresis-augmented fluoride treatment in clinical routines for the control of chronic dentin hypersensitivity.
